# Trial-by-Trial Changes in *a Priori* Informational Value of External Cues and Subjective Expectancies in Human Auditory Attention

**DOI:** 10.1371/journal.pone.0021033

**Published:** 2011-06-16

**Authors:** Antonio Arjona, Carlos M. Gómez

**Affiliations:** Human Psychobiology Lab, Experimental Psychology Department, University of Seville, Seville, Spain; Kyushu University, Japan

## Abstract

**Background:**

Preparatory activity based on *a priori* probabilities generated in previous trials and subjective expectancies would produce an attentional bias. However, preparation can be correct (valid) or incorrect (invalid) depending on the actual target stimulus. The alternation effect refers to the subjective expectancy that a target will not be repeated in the same position, causing RTs to increase if the target location is repeated. The present experiment, using the Posner's central cue paradigm, tries to demonstrate that not only the credibility of the cue, but also the expectancy about the next position of the target are changedin a trial by trial basis. Sequences of trials were analyzed.

**Results:**

The results indicated an increase in RT benefits when sequences of two and three valid trials occurred. The analysis of errors indicated an increase in anticipatory behavior which grows as the number of valid trials is increased. On the other hand, there was also an RT benefit when a trial was preceded by trials in which the position of the target changed with respect to the current trial (alternation effect). Sequences of two alternations or two repetitions were faster than sequences of trials in which a pattern of repetition or alternation is broken.

**Conclusions:**

Taken together, these results suggest that in Posner's central cue paradigm, and with regard to the anticipatory activity, the credibility of the external cue and of the endogenously anticipated patterns of target location are constantly updated. The results suggest that Bayesian rules are operating in the generation of anticipatory activity as a function of the previous trial's outcome, but also on biases or prior beliefs like the “gambler fallacy”.

## Introduction

The attentional function allows the selection of relevant stimuli and appropriate responses. This selection is related to the evaluation of cues and contexts in which the stimuli are inserted. Biasing the neural activity of some percepts would make it possible to produce faster responses if these stimuli appear. Attentional control is particularly important in situations where there are new and complex tasks where the nature of the stimuli and/or responses is uncertain. Preparatory activity based on *a priori* probabilities generated in previous encounters with similar situations would produce a bias for the selection of stimuli and actions adapted to the current context [1]. The counterpart of the preparation process is that it can be correct (valid) or incorrect (invalid); depending on the actual stimulus that appears after preparation, a reduction or an increase in RTs would be expected, respectively.

Posner's central cue paradigm (PCCP) is particularly appropriate for testing congruency between the expected and current stimuli. In this paradigm, the central cue may validly (V trials) or invalidly (I trials) indicate the spatial position of the upcoming target. If the cue is a valid predictor of the target, there is a benefit in the RT with respect to neutral cues. However, if the target is incorrectly cued, a cost occurs in the RT with respect to neutral cues [2], [3]. This effect is termed as a cost-benefit or validity-invalidity effect [2]. This effect would be due not only to the preparation of the sensory cortices related to the predicted spatial location of the target [4], [5], [6], but also partly to preparation for the correct response forvalidly cued target stimuli [7], [5]. Invalidly cued targets would increase their RTs because of the need to reorient the attention and set up the contralateral network to the one preactivated during the preparation period.

An important issue that has scarcely been studied in PCCP is how a correct or incorrect prediction in a given trial can induce changes in the processing of the next trial, i.e. sequential effects. A recent behavioral report on the PCCP addresses this point [8]. These authors found an interaction between the validity in preceding trial and the validity in current trial: the benefits in RTs when compared to neutral cues are greater if a valid trial is preceded by a valid trial (VV trials) rather than an invalid one (IV); but also these authors also found the cost of an invalid trial is greater if it is preceded by a valid trial (VI trials) than by an invalid one (II trials). These results have recently been replicated [9]. We would use the terminology of sequential validity effect to term the interaction between the validity of current and preceding trial. A detailed error analysis has not yet been computed in the sequential validity effect. Other effects related to neutral and catch trials, to the effects of alternate or repeated responses, and to the inhibition of return are also reported by Jongen and Smulders (2007).

These results have recently been interpreted [10] in the sense that PCCP could be considered a very simple form of a cognitive cycle. In this cycle, the preparation, evaluation of the trial, and transfer of information to the next trial would occur sequentially. During the preparation period, the cue would bias the neural set related to the active cue in the direction indicated by the cue, and an *a prior*i probability would be assigned to the cue, determining the amount of attentional resources that would be deployed to the indicated location. The Contingent Negative Variation would be the electrophysiological marker of this preparatory period [5],[6]. During the evaluation period, the valid or invalid nature of the trial would be assessed, with the P3a and P3b being the psychophysiological markers of this cognitive operation [11], [7], [12]. Finally, transfer of information to the next trial would allow a continuous change in the *a priori* credibility that subjects assign to the cues [10]. The credibility would be operationalized as the conditioned probability that, given the cue (S1 stimulus), a target stimulus (S2) would appear in the indicated location (p (S1/S2)). This *a priori* information would be constantly updated, and the P3b component would index the change in this probabilistic value associated with the cue [13], [14]. The key point in the analysis of sequences is that some information can be carried over from one trial to the next. The Bayesian brain perspective fits well with the notion of changing the *a priori* probabilities of the S1–S2 relationship, because it involves the updating of beliefs about subsequent targets based on cues in current and previous trials [15], [16]. In computational terms these ideas are embedded in the Bayesian computational framework proposed by Friston[15], [17], in which neural networks would establish predictions about the credibility they assign to certain environmental cues, and as consequence of the trial outcome the neural networks implicated in this task would change the synaptic weights in order to make better predictions of the cue predictive value in the next future. The changes in early P1 and N1 to attended targets would reflect the confirmation of predicted target (increased precision), while the increase of P3 to invalid targets would reflect the change in the internal model about the precision of the target prediction.

Another issue related to the analysis of sequences is the first-order sequential effect, i.e. the dependency of the response in the current trial on the response in the previous trial. Among the most conspicuous effects are the first-order effects due to the preceding stimulus, whether they are equal to (repetition) or different from (alternation) the preceding stimulus [18]. These first-order effects are dependent on the time elapsed from the current stimulus to the preceding response: the so-called response stimulus interval (RSI). The most common effect is that, for short RSI (less than 500 ms), the reaction time is shorter for repeated stimuli than for alternate stimuli. When the RSI exceeds 500 ms, the repetition effect decreases, and in some cases can become an alternating effect [19]. This differential effect for short and long RSI has been attributed to two different mechanisms [20], [18]. The repetition effect during short RSI could be due to an automatic facilitation. The alternation effect during long RSI can be explained by biasing the probability of which stimulus will be next. The process is controlled by an increased expectancy of the stimulus opposite to the one previously presented. This process is similar to the so called “gambler's fallacy”, where subjects believe that conditioned probability exists between an event and the previous one, when in reality the two events are completely independent. At first glance, the Gamblers fallacy may appear to be a false belief. However, in a changing world, this fallacy may actually be a truthful prior belief that (on average) optimises responses. In short, we may have the prior expectation that things alternate, these sorts of priors have been discussed as an explanation for perceptual switches in bistable perception. Here, we conjecture that subjects believe a priori that targets will appear in alternating locations, at least for two trials sequences (see below).

The study of Event Related Potentials (ERPs) tends to support the view that subjects prepare the response opposite to the one previously executed [21]. In that particular experiment, a tone with a different frequency signaled the response hand. The lateralized readiness response (LRP component) showed that subjects prepared responses corresponding to the hand that had not been used in the previous stimulus [21]; that is, they prepared the alternate response. If the arriving stimulus is the same as the one previously presented, a correction of the movement occurs that is reflected as a change of trend in the LRP component. Similar LRP behavior has been obtained when visual stimuli, rather than auditory tones, are used [5].

However, the sequences of alternation or repetitions would also be able to affect the RTs of subjects as higher order repetition-alternation effects. The most common pattern is of shorter RTs if the higher order pattern is continued, series of repetitions or alternations, than if the pattern is discontinued, i.e. an alternation preceded by series of repetitions [22], [23]. This pattern of RTs could also be included in the framework of the gambler's fallacy if a more broad interpretation of the prior belief is taken, in which it can be assumed that the subjects are looking for certain patterns, series of repetitions or alternations. However it must be remarked that this higher order sequences effects are occurring simultaneously with first-order sequential effects which are the more prevalent effects.

The main aim of the present study was to analyze the sequential effects of S1–S2 trials preceded by other S1–S2s, taking into account the validity/invalidity character of the current and preceding trials. We expect the outcome of the current trial to affect the behavior on the next one. More specifically, in the present report we will try to analyze the sequential dependency of RTs as a function of the validity history of previous trials. Sequences of two and three trials would be analyzed. The hypothesis would be that the validity effect would increase with trial sequence validity because of an increased focused attention on the cued location. In contrast, the invalidity effect would decrease with trialsequence invalidity if less attention is deployed to the cued location. These results would suggest that the deployment of attention would be a function of the validity assigned to the predictive cue as a function of the validity/invalidity history. Not only RTs but also errors should fit this description. Sequences of valid trials should produce not only a decreased reaction time but also an increase in anticipatory responses due to over-preparation. The anticipatory responses would also be expressed as incorrect responses in the invalid trials. An important point about the present experiment is that the cue was visual but the target was an auditory monoaural stimulus, eliminating the possibility that residual eye movements would have any influence on response times or error production.

Additionally, the possibility that a first-order sequential effect would also bias the preparation for the incoming imperative stimuli was tested. For this objective the RTs to the auditory targets when the previous trial differed (Alternation: A), or not (Repetition: R), with respect to the stimulated ear would be compared. The analysis of sequences of Alternation-Alternation (A-A) vs. Repetition-Alternation (R-A) and Repetition-Repetition (R-R) vs. Alternation-Repetition (A-R) were also analyzed in RTs and type of errors. Thesesequenceswere selected to explore the effects on behavior of the confirmation or disconfirmation of repetition and alternation patterns. Furthermore, second-order sequential effect for trials ending in alternation or repetition would be tested to evaluate how prior information about target location can be challenged by experience. The specific hypothesis were: (i) A repeated trial preceded by a repeated trial should have shorter RTs than preceded by an alternation trial (R-R<A-R); and (ii) an alternation trial preceded by an alternation trial should have shorter RTs than preceded by a repeated trial (A-A<R-A).

## Methods

### Participants

Thirty-four subjects (17 female and 17 male, 30 right-handed and 4 left-handed) between 19 and 35 years of age (mean 27) took part in the experiment. The experiments were conducted with the informed and written consent of each subject, following the rules of the Helsinki Convention. The Ethics Committee of the University of Seville approved the study.

### Stimuli and behavioral paradigm

**Figure 1:**
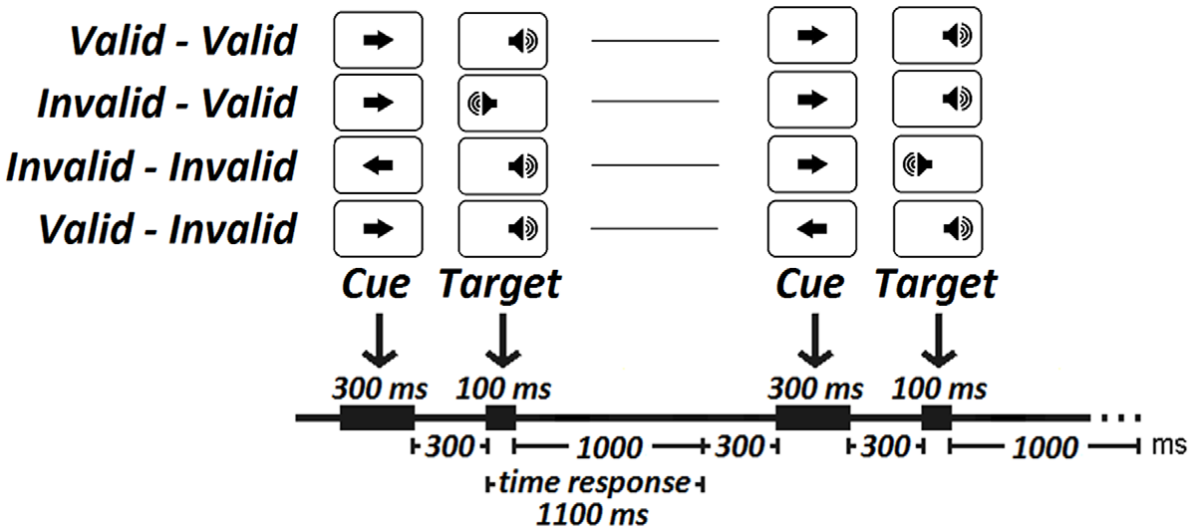
Paradigm for the experiment. The different types of sequence trials (dyads) considered in the experiment are shown. The temporal sequence of stimulus presentation appears in the lower part of the figure. The central arrow was presented in the center of the screen, and the auditory target was presented monoaurally. Notice that the RTs were obtained from the S2 stimulus in the second trial. This corresponds to the stimulus on the right side of the figure in each stimulus sequence.

The stimulus presentation and response recording were computer-controlled (E-Prime 2.0). Participants were seated 60 cm from a computer screen. The subjects participated in a modified version of the PCCP in which the central cues were arrows appearing in the center of the screen, followed by monoaural auditory stimulation. The auditory stimuli were delivered to the subject's ears through headphones. Participants were asked to fix their eyes on a white cross in the center of the screen, and they were instructed to pay attention to the ear signaled by the central arrow, and then press the right button as quickly as possible if the auditory stimulus appeared in the right ear, or the left button if the auditory stimulus appeared in the left ear. The response device was the Cedrus model RB-530. The arrow stimulus was considered the spatial orientation cue, and the monoaural auditory stimulus was the imperative one. The event sequence within a trial was as follows: the central arrow pointer was on for 300 ms (Figure 1), followed by an expectancy period in which a central fixation white cross appeared for 300 ms. Therefore, the total S1–S2 period was 600 ms. The auditory stimulus (1000 Hz) lasted for 100 ms and was randomly presented to the left and right ear with equal probability (0.5). The time of response was 1000 ms., followed by a 300 ms period (Figure 1), producing a total inter-trial interval of 1300 ms.

Each subject was presented with a total of 500 trials divided into five blocks. The central warning stimulus had directional information: in half of the trials it pointed to the right, and in the other half to the left. In 80% of the trials the arrow gave valid information about the target ear (V: valid trials), and in 20% of the trials, the arrow pointed to the ear opposite to where the auditory stimulus would appear (I: invalid trials). The cued location (left or right ear) and the trial Validity or Invalidity were selected randomly. Thus, the experiment presented four types of trials: left valid (LV: 200 trials), right valid (RV: 200 trials), left invalid (LI: 50 trials), and right invalid (RI: 50 trials). It should be noted that left/right in the experimental condition refers to the localization of the auditory stimulus and not the directionality of the warning/arrow stimulus. Therefore, the LI condition refers to preparation of the right side, although the actual target appears in the left ear. The situation is the same for the RI: a left target is indicated by the central cue, but a right target appears. The subjects had to respond to the monaural auditory stimulus with the index finger of the compatible hand. They were informed that the visual cue had an informative value, indicating with high probability the location of the auditory stimulus. RTs and proportion of correct and incorrect responses (responses to the side opposite the stimulated ear), anticipations (responses of targets faster than 180 ms after the auditory target), and omission responses were computed. The percentage of total errors was computed as the sum of all types of errors.

### Behavioral statistical analysis

In the present report, we will focus on the behavioral effects of valid and invalid trials preceded by validly or invalidly cued trials. Therefore we will consider four different types of sequencesof two trials(dyads): valid trials preceded by a valid one (VV) (mean of trials: 316.2 trials, 63.87% of the total, range between different subjects: 310–328); valid trials preceded by an invalid one (IV) (79.7 trials, 16.10% of the total, range: 70–86); invalid trials preceded by a valid one (VI) (79.5 trials, 16.06% of the total, range: 68–87); and invalid trials preceded by an invalid one (II) (19.4 trials, 3.91% of the total, range: 12–29). The different number of trials for different subjects is due to the random selection of trials in each block. In addition, the RTs and errors from sequences of three trials (triads) were computed. The triads were: VVV (250 trials, 51.02% of the total, range: 236–271), IVV (62.8 trials, 12.81% of the total, range: 54–70), VIV (63.2 trials, 12.89% of the total, range: 49–73), IIV (15.7 trials, 3.21% of the total, range: 11–20) III (3.5 trials, 0.71% of the total, range: 0–10), VII (15.7 trials, 3.21% of the total, range: 11–20), IVI (15.9 trials, 3.24% of the total, range: 11–26), VVI (62.9 trials, 12.83% of the total, range: 55–71).

For the dyads of trials, repeated measures ANOVAs for RTs (only for correct responses) and the different types of errors were computed. Three factors were considered: type of sequence (VV, IV, II and VI), side of target presentation (left and right), and whether in the current trial there was alternation or repetition in the position of the target with respect to the previous trial (A vs. R). In the ANOVAs, if a subject presented zero correct responses for any condition, s/he was excluded from the analysis. Our post hoc comparisons were adjusted for multiple comparisons using the Bonferroni correction. We will refer to these as Bonferroni comparisons. The reported p values are already corrected by multiple comparisons.

Repeated measures one-way ANOVA was computed for the statistical analysis of triads. In this case, the triads ending with valid and invalid trials were analyzed separately. Given that our hypothesis was that the deployment of attention would be a function of the trial sequence, only planned *a priori* comparisonswere computed. The comparisons for triads ending with a valid trial were VVV vs. IVV and IVV vs. IIV. The comparisons for triads ending with an invalid trial were III vs. VII and VII vs. VVI. These comparisons were computed for both RTs and Errors.

We also examined alternations of target location in triads. Sequences of Alternation-Alternation (A-A) vs. Repetition-Alternation (R-A), and Repetition-Repetition (R-R) vs. Alternation-Repetition (A-R) were also analyzed in RTs and type of errors by means of a paired t-test.

## Results

**Figure 2:**
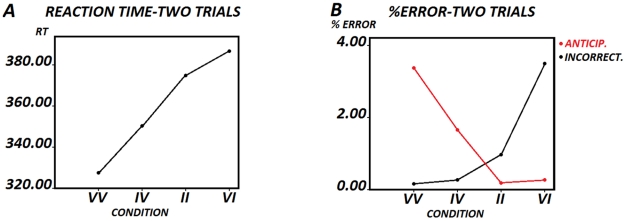
Behavior in the two trial sequences. Fig. 2 Ashows the reaction times in the Valid-Valid (VV), Invalid-Valid (IV), Invalid-Invalid (II) and Valid-Invalid (VI) conditions. Fig. 2B showsthe percentage of anticipatory and incorrect responses in the different sequences. Notice the low percentage of errors and the inverse pattern between anticipatory and incorrect responses.

The ANOVA of RTs of sequences of two trials showed a statistically significant effect of the Alternation-Repetitionfactor (*F [1,31] = 5.319, p<0.028* (mean of Alternation = 357.8166, SD = ±73.32405; mean of Repetition = 365.4573, SD = ±75.38355). The factor type of sequence was also statistically significant (*F [2.155, 66.804] = 48.789, p<0.001*(Figure 2A). No interaction of the effects was obtained. Only 32 subjects were entered in this ANOVA because two of them did not present any correct response for the II condition.

### Analysis of type of sequences

The Bonferroni comparisons contrast showed statistically significant differences between sequences VV-IV (*p<0.006*), VV vs. II (*p<0.006*), VV-VI (*p<0.006*), IV-II (*p<0.006*) and IV-VI (*p<0.006*). The comparison of the II-VI conditions was only significant if the Bonferroni correction was not applied (*p<0.046*), probably because of the low number of trials in the II condition (19.4 trials, 3.91% of the total). The pattern of RTs in the two trials sequences can be seen in Figure 2A.

An error analysis of the two trial sequences was performed. The one-way ANOVA in the different sequences was statistically significant for the total errors (*F [1.517, 47.025] = 7.494, p<0.003*); the anticipations errors (*F [1.118, 34.670] = 7, p<0.010*); and the incorrect responses (*F [2.065, 64.004] = 17.560, p<0.001*). Table 1 shows the mean percentages and standard deviations for the different types of errors. (N = 32). Figure 2B shows the inverse pattern for the percentage of anticipatory and incorrect response errors.

**Table 1:** Percentage of errors: sequences of two trials.

Condition	VV		IV		II		VI	
	Mean	SD	Mean	SD	Mean	SD	Mean	SD
Incorrect	0.16%	0.32	0.27%	0.68	0.97%	3.20	3.49%	2.97
Anticipation	3.38%	6.62	1.65%	4.16	0.18%	1.03	0.27%	0.61
Omission	0.56%	0.83	0.35%	0.80	0.14%	0.84	0.59%	1.54
Total	4.09%	7.28	2.30%	4.55	1.31%	3.38	4.36%	3.75

**Figure 3:**
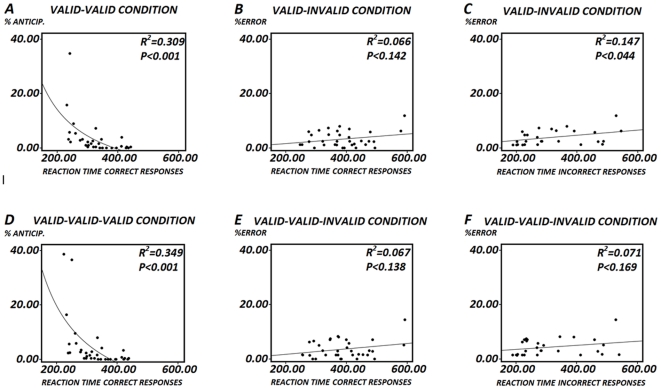
Relationship between errors and reaction times. Fig. 3A shows the relationship of the correct response RTs with the percentage of anticipation errors in the valid-valid condition. This relationship was modeled as an inverse equation by means of a polynomial fit. Fig. 3B shows the relationship between the RTs of correct responses and the percentage of incorrect responses in the invalid-valid condition. Fig. 3C shows the relationship between the RTs of incorrect responses and the percentage of incorrect responses. If graphs 3B and 3C are compared, faster RTs of incorrect responses with respect to RTs of correct responses can be observed. Figs. 3D, 3E and 3F show the same information as 3A, 3B and 3C, but for the valid-valid-invalid sequence. Also notice that only the data in Figs. 3A and 3D can be fitted by an inverse relationship.

The Bonferroni comparisons contrast showed statistically significant differences for incorrect responses between VV-VI (*p<0.006*), IV-VI (*p<0.006*) and II-VI (*p<0.006*); for anticipations between VV-IV (*p<0.018*) and VV-VI (*p<0.042*); and for total errors between VV-IV (*p<0.030*), VV-II (*p<0.030*), IV-VI (*p<0.054*) and II-VI (*p<0.006*). Figure 3A shows the relationship of the correct response RTs with the percentage of anticipations. This was an inverse relationship, indicating that faster subjects also produced the largest number of anticipations.

An additional comparison was made between the RTs of the incorrect responses in the VI condition and those of the correct responses in the same condition. This comparison was made to check whether the incorrect responses were due to very fast responses in which not enough auditory information was gathered. The repeated measures t-test showed a statistically significant difference in correct vs. incorrect responses in the VI condition (*F [1,27] = 21.730, p<0.001*; mean of RTs incorrect responses = 387.1751, SD = ±89.6058; mean of RTs in incorrect responses = 319.3455, SD = ±113.1817) (N = 28). Given the low number of incorrect responses, six subjects did not show any incorrect response and were not included in the analysis. Figure 3B shows the relationship between the RTs of correct responses and the percentage of incorrect responses. Figure 3C shows the relationship between the RTs of incorrect responses and the percentage of incorrect responses. If graphs 3B and 3C are compared, the faster RTs of incorrect responses with respect to RTs of correct responses can be observed. Another additional point of VI trials is that the percentage of incorrect responses did not show an inverse relationship with RTs (as in figure 3A), suggesting that these errors are not exclusively due to anticipatory behavior.

**Figure 4:**
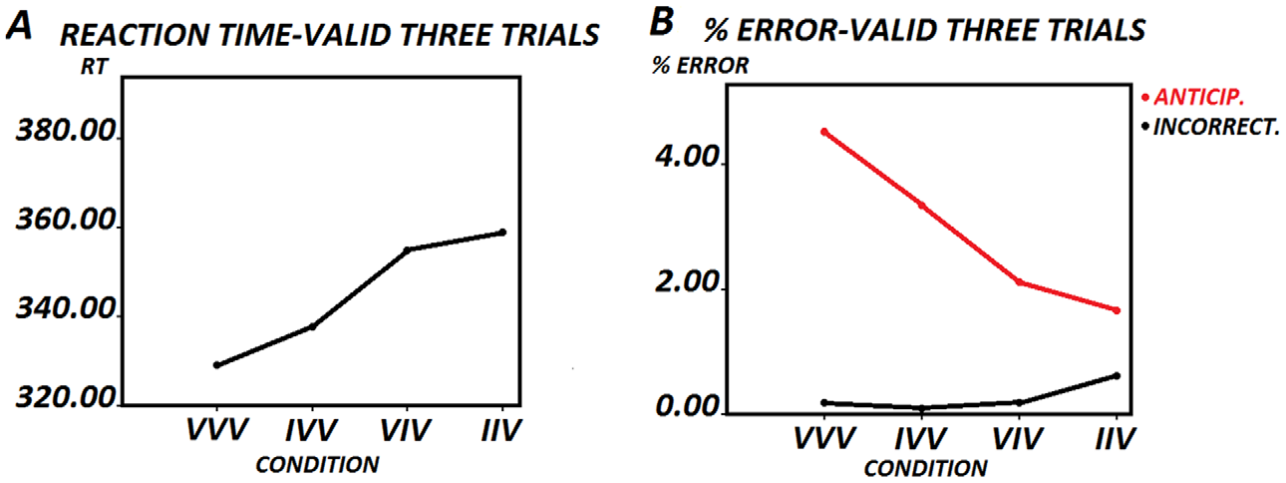
Behavior in the three trial sequences (triads) ending in a valid trial. Fig. 4A shows the reaction times in the Valid-Valid-Valid (VVV), Invalid-Valid-Valid (IVV), Valid-Invalid-Valid (VIV) and Invalid-Invalid-Valid (IIV) conditions. Fig. 2B shows the percentage of anticipatory and incorrect responses in the different sequences. Notice the low percentage of errors and the inverse pattern between anticipatory and incorrect responses.

The ANOVA of the reaction times of sequences with three trials ending in a valid trial (VVV, IVV, VIV, IIV) showed a statistically significant effect for the *type of sequence* factor (*F [1.759, 58.052] = 22.772, p<0.001* (Figure 4A)(N = 34). The reason there are 34 subjects in the triad analysis, while in the dyads there are only 32, is that in triads the left and right target conditions were collapsed. The planned Bonferroni comparisons contrast showed statistically significant differences between sequences VVV-IVV (*p<0.002*) and IVV-IIV (*p<0.002*). (N = 34). Figure 3D shows the relationship between the correct response RTs and the percentage of anticipations. This was an inverse relationship, indicating that faster subjects are also those producing a greater number of anticipations. The errors from the trial sequences ending with a valid trial were analyzed. The one-way ANOVA was statistically significant only for the total errors in the different sequences (*F [1.350, 44.555] = 4.459, P<0.030*). Table 2 and Figure 4B shows the mean percentages and standard deviations. (N = 34). The planned Bonferroni comparisons showed statistically significant differences for total errors between VVV-IVV (*p<0.016*), but not between IVV-IIV (*p<0.404*).

**Table 2:** Percentage of errors: sequences of three trials ending in a valid trial.

Condition	VVV		IVV		VIV		IIV	
	Mean	SD	Mean	SD	Mean	SD	Mean	SD
Incorrect	0.18%	0.34	0.09%	0.38	0.19%	0.67	0.61%	2.05
Anticipation	4.50%	9.16	3.33%	6.67	2.11%	5.14	1.64%	3.57
Omission	0.63%	0.96	0.24%	0.72	0.36%	0.94	0.16%	0.95
Total	5.32%	9.91	3.67%	7.21	2.67%	5.36	2.42%	4.11

**Figure 5:**
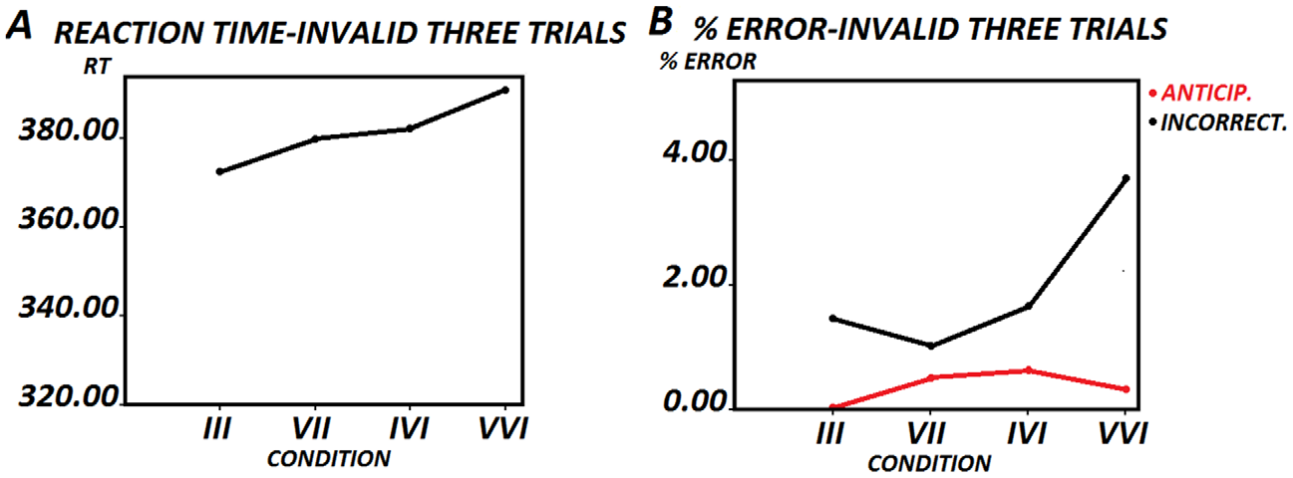
RTs in the three trial sequences (triads) ending in a valid trial. Fig. 5A shows the reaction times in the Invalid-Invalid-Invalid (III), Valid-Invalid-Invalid (VII), Invalid-Valid-Invalid (IVI) and Invalid-Invalid-Valid (IIV) conditions. Fig. 2B shows the percentage of anticipatory and incorrect responses in the different sequences.

The ANOVA of the reaction times for sequences with three trials ending with an invalid trial (III, VII, IVI, VVI) did not show a statistically significant effect for the factor *type of sequence* (*F [1.506, 45.166] = 1.600, p<0.216* (Fig. 5A). (N = 31; because there were 3 subjects who did not present any case in the III condition). The errors from three trial sequences ending with an invalid trial were also analyzed. The one-way ANOVA was statistically significant only for the total errors in the different sequences (*F [1.911, 63.076] = 3.785, p<0.030*). Table 3 and Figure 5B show the mean percentages and standard deviations. (N = 34). Finally, the planned Bonferroni comparisons showed a statistically significant difference for total error between VII-VVI *(p<0.002)*, but not between III-VII *(p<1.626)*.

**Table 3:** Percentage of errors: sequences of three trials ending in a invalid trial.

Condition	III		VII		IVI		VVI	
	Mean	SD	Mean	SD	Mean	SD	Mean	SD
Incorrect	1.47%	8.57	1.03%	3.15	1.66%	3.46	3.71%	3.38
Anticipation	0%	0	0.52%	2.19	0.65%	2.15	0.34%	0.90
Omission	0%	0	0.19%	1.14	0.44%	1.80	0.61%	1.90
Total	1.47%	8.57	1.75%	3.78	2.75%	4.67	4.67%	4.04

An additional comparison was made between the RTs of the incorrect responses in the VVI condition and those of the correct responses in the same condition. The repeated measures t-test showed a statistically significant difference in correct vs. incorrect responses in the VVI condition (*F [1,27], p<0.001*; mean of RTS of correct responses = 392.0075, SD = ±90.2392; mean of RTs of incorrect responses = 313.8533, SD = ±113.6903). Given the low number of incorrect responses, six subjects did not show any incorrect response and were not included in the analysis. Figure 3E shows the relationship between the RTs of correct responses and the percentage of incorrect responses. Figure 3F shows the relationship between the RTs of incorrect responses and the percentage of incorrect responses. If graphs 3B and 3C, and 3D and 3F, are compared, it can be seen that the RTs of incorrect responses are faster than the RTs of correct responses.

### Analysis of thefirst-order and second-ordersequential effects of the Alternation and Repetition factor

The errors for the Alternation and Repetition factorwere analyzed. The one-way ANOVA was statistically significant only for the incorrect responses (*F [1,31] = 10.847, p<0.002*). Table 4 shows the mean percentages and standard deviations. (N = 32).

**Table 4:** Percentage of errors: first order repetition-alternation effects.

Condition	A		R	
	Mean	SD	Mean	SD
Incorrect	0.53%	0.69	1.00%	0.75
Anticipation	1.86%	3.02	1.54%	3.02
Omission	0.29%	0.47	0.36%	0.43
Total	2.69%	3.47	2.91%	3.50

**Figure 6:**
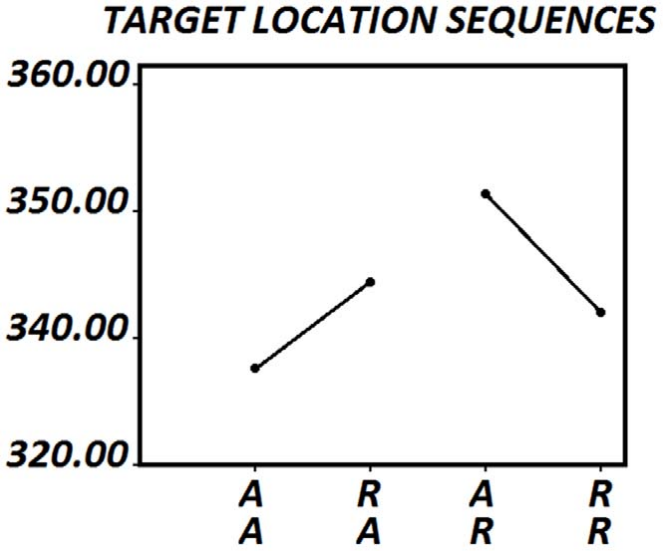
RTs to second-order alternation repetition effects. A-A: sequences of two alternations in target locations (i.e. left-right-left). R-A: Sequences of repetition and alternation of target location (i.e. left-left-right). A-R: Sequences of alternation and repetition of target location (i.e. left-right-right). R-R: Sequences of repetition and repetition of target location (i.e. left-left-left).

As previous results indicated faster RTs in the Alternation condition than in the Repetition condition, the possibility that a confirmatory hypothesis would also be acting to modulate the RT was checked by means of a comparison of the A-A vs. R-Aand R-R vs. A-R sequences. If subjects presented a false belief of the gambler's fallacy type, A-A sequences would imply a confirmation of this belief, and RTs should be faster than in the R-A sequences, where this belief was disconfirmed in the previous trial. Results for RTs are presented in Figure 6 and errors are presented in Table 5. The same argument stands for second order sequential effects in which the last trial is a repetition. The paired t-test showed that there was a decreased RTs in the R-R condition with respect to the A-R condition, (*p<0.001*) (Figure 6). Furthermore, there was a statistically significant higher number of incorrect responses in the condition A-R than in the R-R condition(*p<0.007*).

**Table 5:** Percentage of errors: second order repetition-alternation effects.

Condition	A-A		R-A		R-R		A-R	
	Mean	SD	Mean	SD	Mean	SD	Mean	SD
Incorrect	0.33%	0.65	0.49%	0.59	0.43%	0.84	1.08%	0.96
Anticipation	1.98%	3.38	1.53%	2.52	1.66%	2.59	1.09%	2
Omission	0.25%	0.46	0.25%	0.57	0.28%	0.47	0.31%	0.49
Total	2.57%	3.39	2.29%	2.60	2.39%	2.7	2.48%	2.37

## Discussion

The results indicate an increase in RT benefits when dyads and triads of valid trials occurred. The analysis of errors indicates an increase in anticipatory behavior that grows in VV sequences compared to IV and VI sequences. There was a statistical trend of increased costs in the dyads of II trials with respect to invalid trials preceded by valid trials (VI). The analysis of errors showed an increase in incorrect responses in sequences ending in invalid trials, while anticipatory responses were very low in the VI and II trials. On the other hand, there was also a benefit in RTs and a reduced number of incorrect responses when a trial was preceded by trials in which the position of the targets was different from that of the current trial(first-order alternation effect). Furthermore RTs of A-A trial sequences were faster than R-A sequences and R-R were faster than A-R. Taken together, these results suggest that in central PCCP, the anticipatory activity, the validity-invalidity effect, and the alternating effect are modulated by the previous trial sequences. This sequential modulation has two independent sources: (i) a previous-trial validity dependent preparatory activity and (ii) a previous-target location dependent preparatory activity. The results suggest that Bayesian rules tend to operate in generating anticipatory activity based on confirmatory outcomes of explicit cues, but also based on confirmatory outcomes of priors, as in the “gambler's fallacy.

### Sequential validity effects

In the present experiment, the trials ending with valid conditions (VV and IV) were faster than the trials ending with invalidly cued targets (VI, II), fitting the classically described cost-benefit pattern of the PCCP [2]. The current theory on how valid cueing is able to decrease reaction times is based on data suggesting that directional cues activate the opposite sensory cortex to the signaled hemifield, facilitating perceptual activities once the sensory stimulus arrives [11], [4]–[6]. Another source for facilitating responses to valid cues would be the anticipatory neural activity in motor and premotor cortices needed for the response to the expected target [5]. In this way, attention during the PCCP can be related to the idea of Bayesian inference, in the sense that the subject is making predictions about the possible position of the target, inducing a pre-activation of the areas supposedly needed for the next incoming target. This framework makes it possible to explain not only benefits, but also RT costs, given that the whole network must be reorganized when an invalidly cued target is presented. These ideas also fit the biased competition model [1], given that a central executive would make it possible to boost activity in selective sensory cortices related to the predicted perception, favoring its perception over any other competing stimulus. The dorsolateral fronto-parietal network would be the key attentional structures feeding the sensory cortices with neural inputs that would increase the gain in the predicted positions [24]. For invalidly cued targets, the right inferior frontal gyrus would be one of the key areas participating in denoting the novel character of the target [25], [24].

However, the main objective of the present report is related to the sequential validity effect [8], [9]. As indicated above, the targets in the last trials in sequences of VV and VVV trials correspond to the dyads' and triads' fastest RTs conditions, but they are also the conditions with the highest number of anticipations, indicating that, in part, the increased RTs correspond to hand movements without enough available information. However the low number of errors make difficult to assume that all the sequential validity effect is due to pure anticipatory responses, rather than to preparatory attention. The pattern of RTs follows the rule of VV<IV<II<VI. The pattern of II<VI is particularly important to support the idea of preparatory attention for the sequential validity effects [4]–[6]. This result suggest that if an invalid trial is preceded by an invalid trial the subjects deployed less attention to the indicated ear, and responses are faster than in VI trials, in which more credibility is assigned to the cue and responses are consequently delayed. The RTs patterns for triads suggest also a trial-by-trial change of the intensity of deployed attention. The patterns for the triads corresponds to a statistically significant pattern of VVV<IVV<IIV, and a qualitative pattern of III<VII<VVI (probably not statistically significant due to the low number of trials).

The pattern of errors is an increased number of anticipations in the VV sequences, and an increased number of incorrect responses in the VI conditions, while the anticipation remains very low in the VI condition. Moreover, incorrect responses are faster than correct responses in VI (and VVI) sequences. Interestingly, while the relationship between percentage of anticipations and RTs was an inverse relationship (Fig. 3A), the same relationship did not occur with incorrect responses (figs 3B and 3C). These results indicate that incorrect responses are too-fast responses in which not enough auditory information has been gathered, but they are not purely anticipatory as in the VV condition, indicating that a trial being preceded by a valid trial generates anticipatory activity that, in general, is not enough to trigger a movement, but that can increase the number of errors. Therefore, attentional bias in the sequences is observed as anticipatory in VV trials and as incorrect responses in VI trials. It is possible that there is a response latency time in which sensory information is gathered, thus influencing behavior, but the responses are so fast that a weighting average of the exogenous information with the endogenous information occurs. This interaction between endogenous (anticipatory) and exogenous (sensory) activity has been proposed for the express saccades [26]. The express saccades is an ideal paradigm for studying this “intertidal” period because in the superior colliculus there is vectorially weighted predictive and visual coding, producing saccades whose precision amplitude errors, measured in visual angle degrees, have an inverse relationship with latency time [26]. In the experiment reported here, the anticipatory behavior in VV condition would be a synergy between the prediction and the sensory information, while the incorrect responses in VI condition would reflect the incongruency between prediction and the actual stimulus. The present report contains enough quantitative description of the experimental results in order to produce a mathematical modeling of the RTs an errors pattern of these sequential analysis. Therefore, the suggestion of a response inter-tidal period in which information that a target is present producing anticipatory responses in VV trials and incorrect fast responses in VI trials remains to be modelized. This inter-tidal period would be similar to the intermediate phase (responses between 200–300 ms) of incompatible noise trials in the “noise-compatibility paradigm”, in which the presence of incompatible letters activate the incorrect response producing more errors than for long latency responses (more than 300 ms) which would be more accurate [27].

The patterns obtained for RTs and errors suggest that information is being transferred from one trial to another, so that a confirmation of the explicit hypothesis about the position of the next target encoded by the cue is transferred to the next trial and, consequently, influences the level of attention. This argument is related to the proposal by Yu and Dayan (2005) [28], when analyzing the cost-benefit pattern, in which they highlight the balancing of the relative influence of bottom-up sensory information and top-down prior expectations by weighting them according to their relative precision (credibility). Indeed, it has been proposed that attention can be understood purely in terms of optimizing the precision or credibility of representations during hierarchical inference in the brain [16]. Therefore, one possible explanation for the longer RTs in the IV condition than in the VV condition (and VI with respect to II) would be continuous updating of the predictive value subjects assign to the spatial cue. Yu and Dayan (2005) [28] proposed that PCCP is a good example of how probabilistic Bayesian learning occurs. In trials in which expectations are violated, the subjects would pay less attention to top–down signals (cues) and more attention to bottom–up processes (target stimuli). In other words, the cue's predictive value would change on a trial-by-trial basis. This value would be lower in the IV condition than in the VV condition, consequently producing longer RTs in the former than in the latter. The same concept applies to the comparison of the lower RTs obtained in the VVV condition with respect to the IVV condition. It must be noted that a comparison of the VV and IV conditions would reflect a local effect of the outcome of the previous trial, superimposed on the more robust cost–benefit effect due to global contingencies on the task and the implicit spatial value of the cues [2]. In the same sense, the II trials would be faster than the VI trials because attention would be more related to bottom-up processes in the II condition that in the VI condition.

One central issue pertaining to Bayesian inference is that when a target is encountered, the validity of the prediction (prediction error) must be computed, and the credibility or precision of the hypothesis about where the target should appear as a function of the directional cue must be updated, producing consequences in the next trial. The present results on RTs and errors in dyadic and triadic sequences, with decreased RTs and increased anticipatory errors, and the results from Jongen and Smulders (2007) on RTs in dyadic sequences, fit the idea of Bayesian inferences during the PCCP. This framework supports the idea that the PCCP is a good example of a cognitive cycle in which preparation for targets, evaluation of trial outcome, and transferring of information from the current trial to the next trial make up a cognitive cycle that facilitates adaptation to environmental cues [29],[10].

Another possible explanation of the sequential validity effect would be in terms of increased strategic or cognitive control in V trials after an I trial occurred, i.e. more cognitive control in IV trials than in VV trials [30], [31]. However this explanation would have difficulties to explain why the II condition is faster than the IV condition. The II<IV result obtained in present report has also been obtained in several reports [8], [12]. In fact, under the cognitive control hypothesis it should be expected the opposite result, more cognitive control after two subsequent Invalid trials (II) than following only one invalid trial (IV condition). The experiments in which increased cognitive control has been proposed to explain longer RTs after incongruent trials had shorter ISIs than the PCCP, and also no cue was interposed between two target stimuli [27], [32]–[35].

The lower RTs and errors in trials preceded by trials with a different auditory stimulus position indicate that an alternation effect [19] appears in this sequence. The lack of interaction between the type of trial (VV, IV, II, and VI) and the position change factor suggests that the expectancy linked to the alternation effect is exclusively based on the position of the previous target. This previous target alternation effect is particularly interesting, given that it operates independently of the type of trial in which it is embedded, and seems to overcome the fact that a directional cue is inserted between the two targets. Given that there are 1900 ms between the current and previous trials, the alternation effect obtained can be included in the type of sequential effects in which the expectancy of next target is computed [20], [18]. This expectancy would follow a rule similar to the gambler's fallacy, where subjects have the tendency to think that the occurrence of a phenomenon makes the occurrence of this same phenomenon less likely in the next trial. For instance, if the previous trial presented a left target, the subject would have a certain tendency to think that the next trial would be right, independently of the type of previous trial. This phenomenon has been studied, the so-called alternation effect, and seems to depend on motor activation as indexed by the Lateralized Readiness Potential [21], [36].

One important consequence of the present results is that this prior can be challenged by experience. In the present experiment, the A-A sequence, which confirms the sequences of alternations, is faster than the R-A sequences in which the sequential repetitionwas contradicted in currenttrial [22], [23]. Similarly, the R-R sequence in which the pattern of target location repetitions is confirmed is faster than the A-R in which the sequential alternation is contradicted in current trial. This result implies that the “gambler's fallacy” in control subjects without any specific cognitive problems, if an interpretation of this belief as looking for alternation or repetition patterns is done, can be modified by experience in a Bayesian form. Unlike in the present study, Jongen and Smulders (2007) did not find an alternation effect. This difference could be due to the fact that the alternation effect obtained here is rather small, but still statistically significant, probably due to the high number of experimental subjects.

Finally, it should be mentioned that the cost-benefit pattern is induced by the cue [2], but some modulation occurs depending on the history of the sequence in which a given trial is embedded. Basically, this sequence would modulate the preparation for the next trial following (i) a Bayesian rule which updates the credibility of the cue [15], [16], [9] and (ii) a small influence of the gambler's fallacyprior belief confirmation or disconfirmation. Therefore, confirmatory outcomes in fast reaction times experiments take into account explicit cues (cost-benefit pattern of valid and invalid trials), sequential validity effect(faster if previous trials presented a confirmatory outcome), an alternation effect based on the expectancy that targets different from the previous one are more probable, and the endogenous search for repetitions or alternations patterns. The sequential modulating effects are well explained in the Bayesian brain hypothesis framework [15], [16].
